# Supplementary material for: Loss of microglial MCT4 leads to defective synaptic pruning and anxiety-like behavior in mice
Source: Nat Commun. 2023 Sep 16;14:5749. doi: 10.1038/s41467-023-41502-4 (PMC10505217; doi:10.1038/s41467-023-41502-4)

Supplementary Figures

a

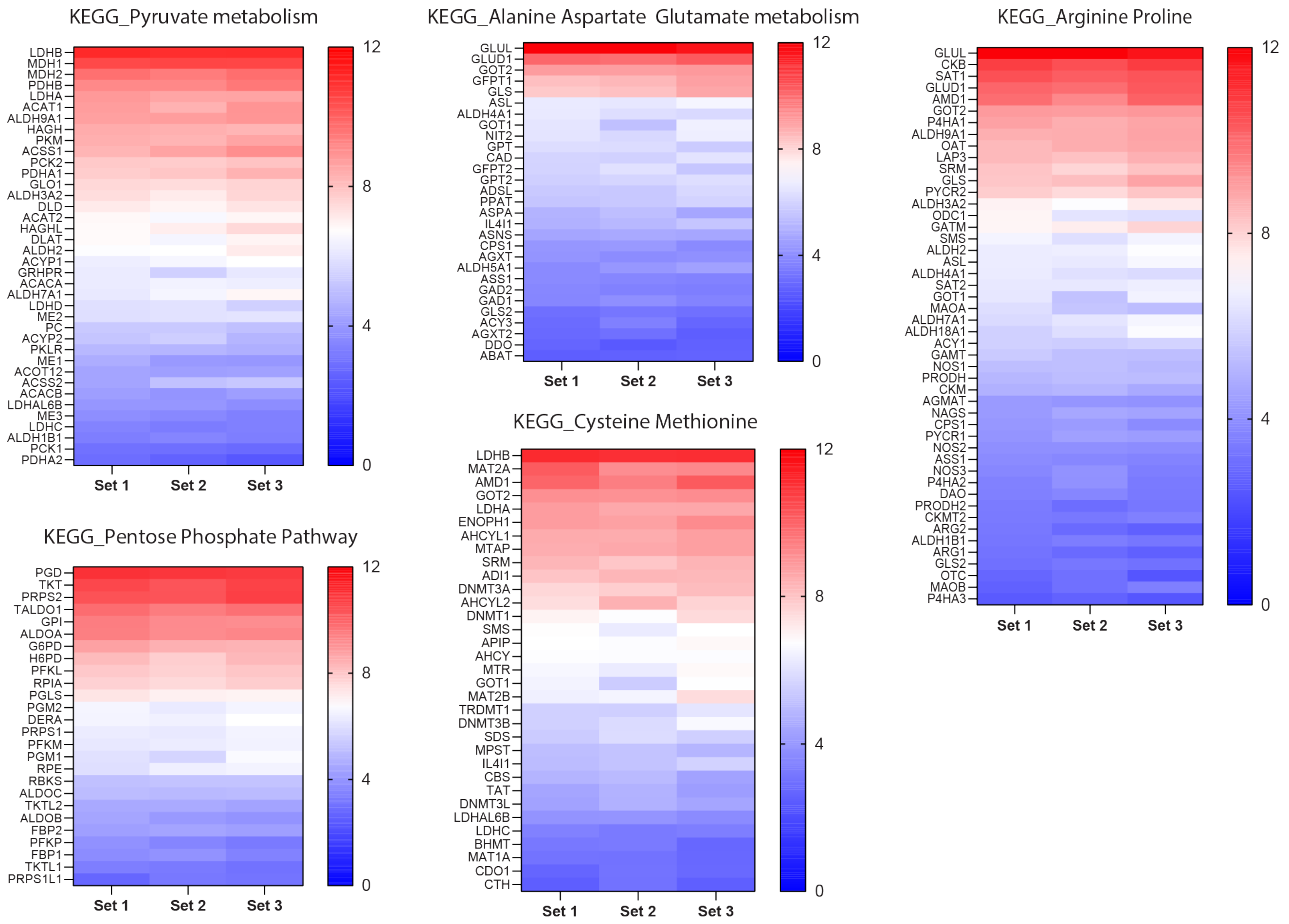

b

Metabolic pathway	Microglia_compared_to	P-value
KEGG_PENTOSE_PHOSPHATE_PATHWAY	Astrocytes	0.500405
KEGG_PENTOSE_PHOSPHATE_PATHWAY	Oligodendrocytes	1.11E-18
KEGG_PENTOSE_PHOSPHATE_PATHWAY	Neurons	3.98E-23
KEGG_ALANINE_ASPARTATE_AND_GLUTAMATE_METABOLISM	Astrocytes	8.64E-41
KEGG_ALANINE_ASPARTATE_AND_GLUTAMATE_METABOLISM	Oligodendrocytes	8.62E-54
KEGG_ALANINE_ASPARTATE_AND_GLUTAMATE_METABOLISM	Neurons	4.43E-06
KEGG_PYRUVATE_METABOLISM	Astrocytes	0.227298
KEGG_PYRUVATE_METABOLISM	Oligodendrocytes	1.27E-17
KEGG_PYRUVATE_METABOLISM	Neurons	4.66E-37
KEGG_FATTY_ACID_METABOLISM	Astrocytes	3.75E-32
KEGG_FATTY_ACID_METABOLISM	Oligodendrocytes	8.11E-06
KEGG_FATTY_ACID_METABOLISM	Neurons	7.32E-35
KEGG_OXIDATIVE_PHOSPHORYLATION	Astrocytes	1.01E-208
KEGG_OXIDATIVE_PHOSPHORYLATION	Oligodendrocytes	9.00E-170
KEGG_OXIDATIVE_PHOSPHORYLATION	Neurons	0
KEGG_GLYCOLYSIS_GLUCONEOGENESIS	Astrocytes	1.85E-29
KEGG_GLYCOLYSIS_GLUCONEOGENESIS	Oligodendrocytes	1.35E-139
KEGG_GLYCOLYSIS_GLUCONEOGENESIS	Neurons	9.95E-120

Supplementary Figure 1: Microglial transcriptome indicates high potential for metabolic flexibility.

(a) Transcriptional basis for microglia metabolic flexibility. Heatmaps of the microglial expression levels of genes annotated within substrate metabolism-related KEGG-pathways. Data were obtained from the dataset published in Pinto et al. 2012[26], from three independent experiments, shown as normalized intensity values. **(b)** Exact p-values calculated with Wilcoxon statistical test (two-tailed) for all comparisons between hippocampal cell populations, as shown in Figure 1b.

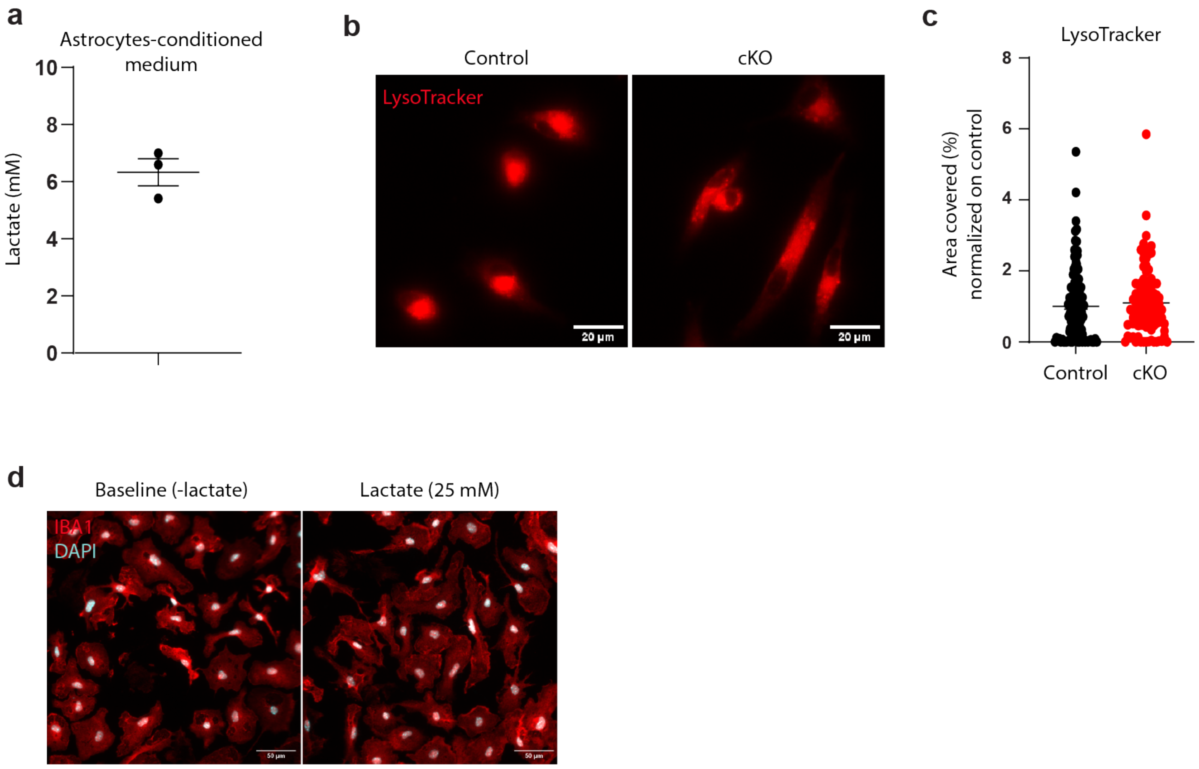

Supplementary Figure 2: Microglia are not stressed upon lactate treatment and extracellular lactate drives the lysosomal differences observed in presence or absence of MCT4

(a) Lactate content in astrocytes-conditioned medium, with 24h conditioning. Lactate concentration (mM) was measured taking advantage of an enzymatic-spectrophotometric method, as described by Rosenberg and Rush[35]. **(b)** Representative z-stack projections of LysoTracker staining in control and cKO primary microglia cultured in lactate-free medium (scale bar: 20µm) and **(c)** relative quantification of area covered by LysoTracker signal, normalized to controls. Data points represent individual cells from N=2 independent experiments. Control: n=52 cells; cKO: n=52 cells. **(d)** Representative images of primary microglia (IBA1+) at baseline or supplemented with sodium lactate (25mM) for 24h; Scale bar: 50µm. The purity of the cultures was at least 95%. No major morphological changes nor cellular stress hallmarks were observed across conditions.

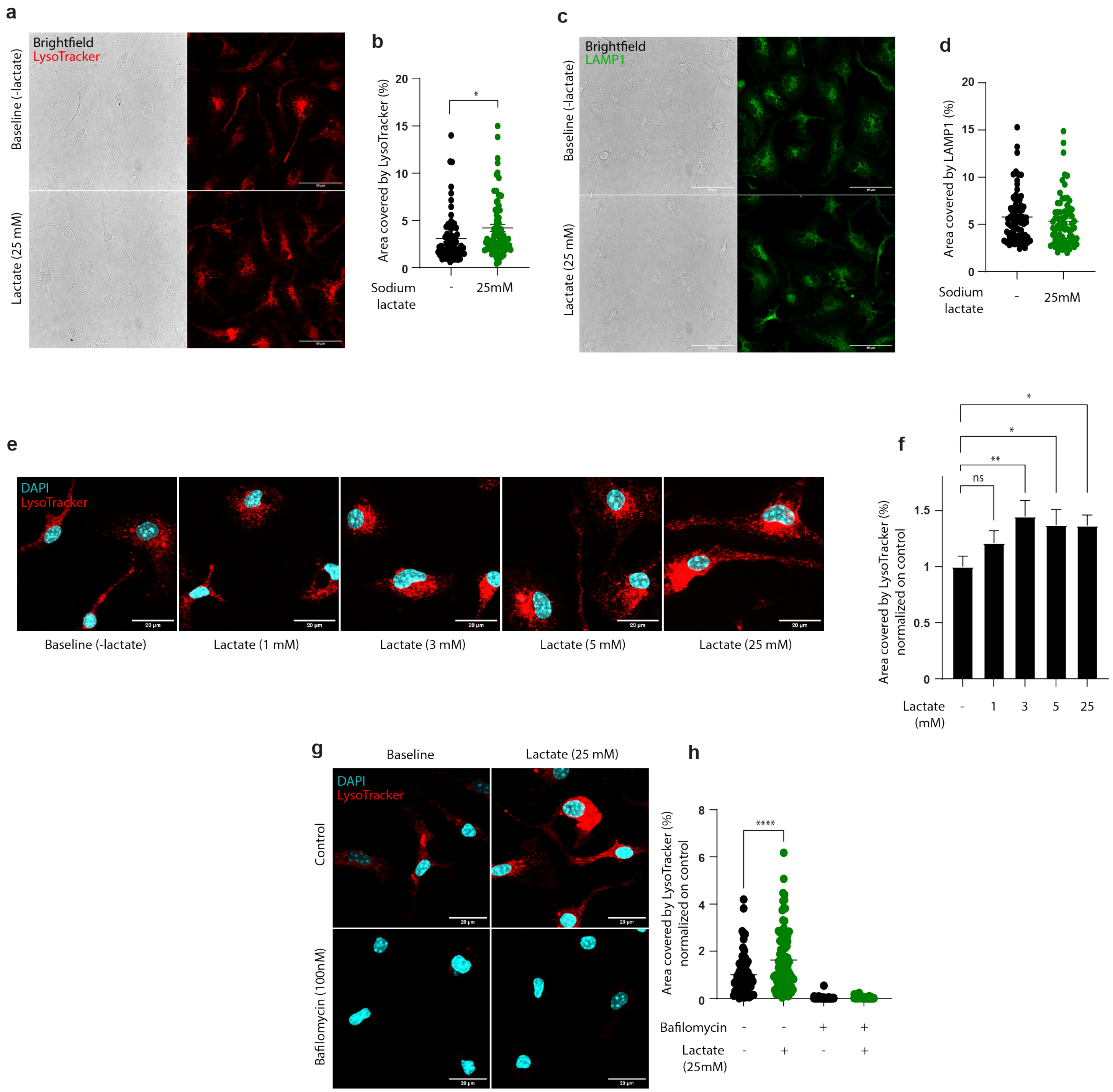

Supplementary Figure 3: Lactate-driven lysosomal acidification in microglia is dose- and vATPase-dependent, and it is not accompanied by alterations in the LAMP1+ vesicles pool

(a) Representative confocal z-stack projections of LysoTracker labelling in wild-type primary microglia, treated with control medium or 25mM lactate-containing medium. Scale bar: 50μm. **(b)** Quantification of area covered by LysoTracker in control or lactate-treated primary microglia. Each data point represents

an individual cell, control: n=66 cells; lactate 25mM: n=70 cells from N=2 independent experiments. Two-tailed unpaired t-test. *p=0.0254. **(c)** Representative confocal z-stack projections of LAMP1+ structures in wild-type primary microglia, treated with control medium or 25mM lactate-containing medium. Scale bar: 50µm. **(d)** Quantification of area covered by LAMP1 in control or lactate-treated primary microglia. Each data point represents an individual cell, control: n=75 cells; lactate 25mM: n=70 cells from N=3 independent experiments. **(e)** Representative confocal z-stack projections and **(f)** relative quantification of LysoTracker labelling in wild-type primary microglia, treated with control medium or increasing concentration of lactate. Scale bar: 20µm. Data points represent individual cells from N=2 independent experiments. Baseline (-lactate): n=51 cells, Lactate (1mM): n=58 cells; Lactate (3mM): n=59 cells; Lactate (5mM): n=58 cells; Lactate (25mM): n=66 cells. One-way ANOVA, uncorrected Fisher LSD test. **p(control:lactate3mM) = 0.0097; *p(control:lactate5mM) = 0.0326; *p(control:lactate25mM) = 0.0301. **(g)** Representative confocal z-stack projections and **(h)** relative quantification of LysoTracker labelling in wild-type primary microglia, treated with control medium or 25mM lactate-containing medium in presence or absence of bafilomycin (100nM, vATPase inhibitor). Scale bar: 20µm. Each data point represents an individual cell, Control-Baseline: n=65 cells; Control-lactate 25mM: n=91 cells; Bafilomycin-Baseline: n=75 cells; Bafilomycin-lactate 25mM: n=71 cells from N=2 independent experiments. Two-tailed unpaired t-test. ****p < 0.0001.

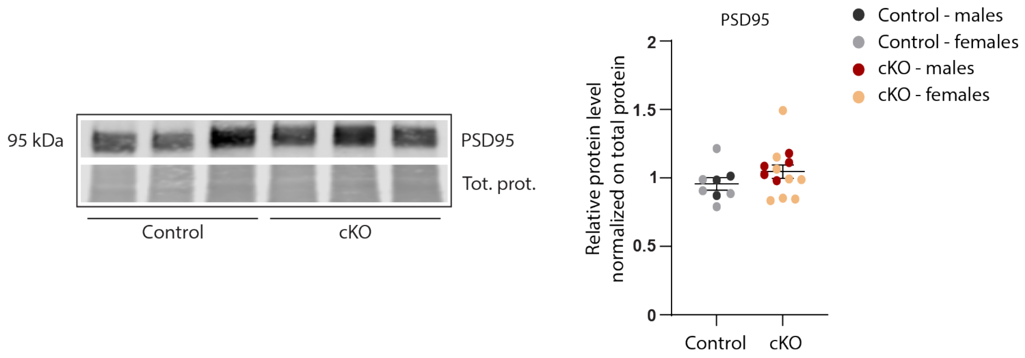

Supplementary Figure 4: PSD95 protein content is not altered in the cKO hippocampus at P15

Representative PSD95 western blots of hippocampal homogenates from P15 control and cKO mice and relative quantification. normalized to the control group. Each data point corresponds to one animal. Control: N=3 males; N=5 females; cKO: N=5 males; N=8 females. Two-tailed unpaired t-test.

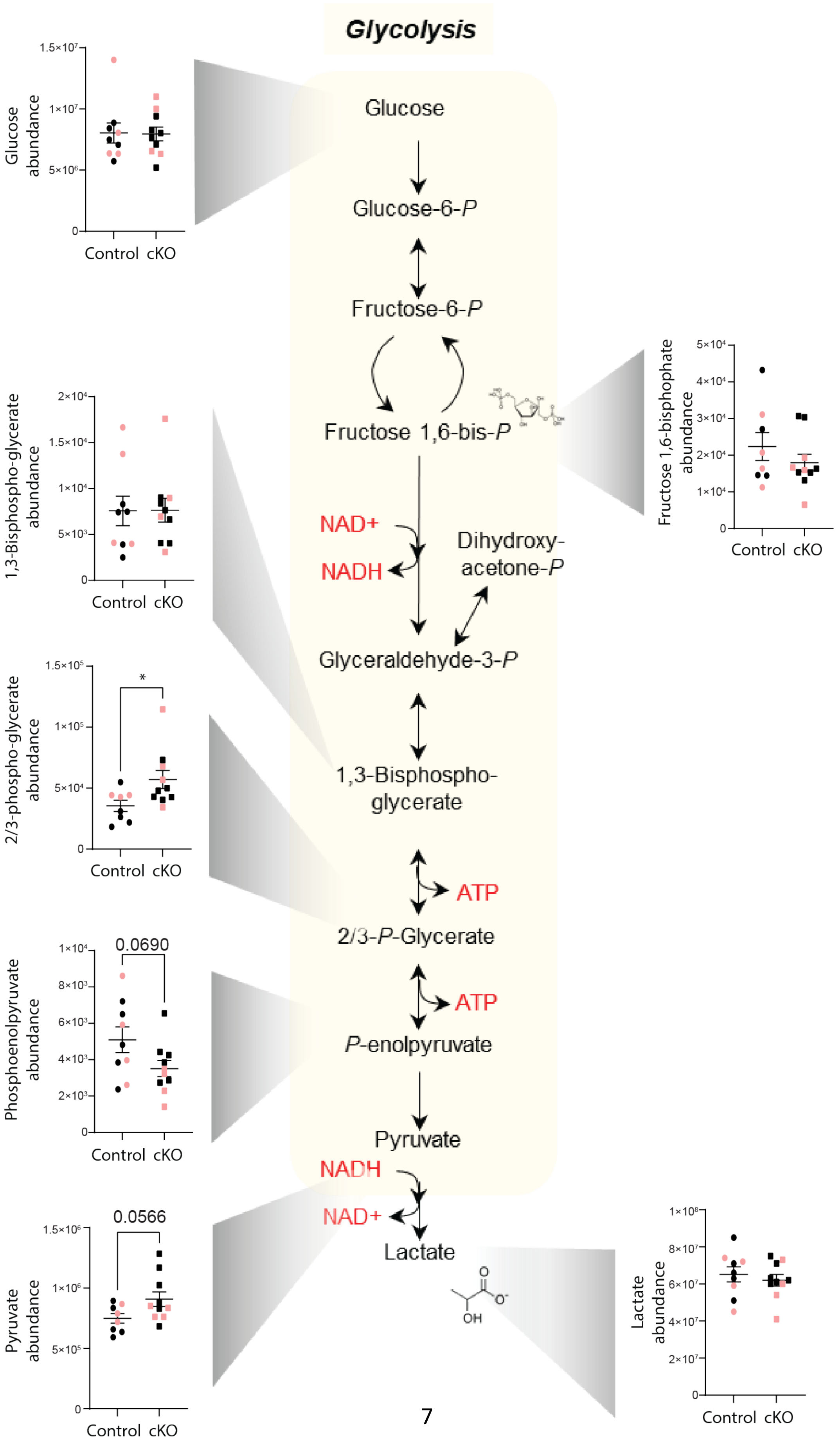
Glycolysis
Glucose
Glucose-6-P
Fructose-6-P
Fructose 1,6-bis-P
NAD+
NADH
Dihydroxy-acetone-P
Glyceraldehyde-3-P
1,3-Bisphospho-glycerate
ATP
2/3-P-Glycerate
ATP
P-enolpyruvate
Pyruvate
NADH
NAD+
Lactate
Glucose abundance
Control cKO
Fructose 1,6-bisphophate abundance
1,3-Bisphospho-glycerate abundance
2/3-phospho-glycerate abundance
*
Phosphoenolpyruvate abundance
0.0690
Pyruvate abundance
0.0566
Lactate abundance

Supplementary Figure 5: Loss of microglial MCT4 induces changes in the abundance of some glycolysis intermediates in the P15 hippocampus

Abundance of glycolysis metabolites measured via LC-MS in the hippocampus of P15 mice. Controls: N=5 males; N=4 females. cKO: N=6 males; N=4 females. *p(2/3-phospho-glycerate)=0.0339; two-tailed unpaired t-test.

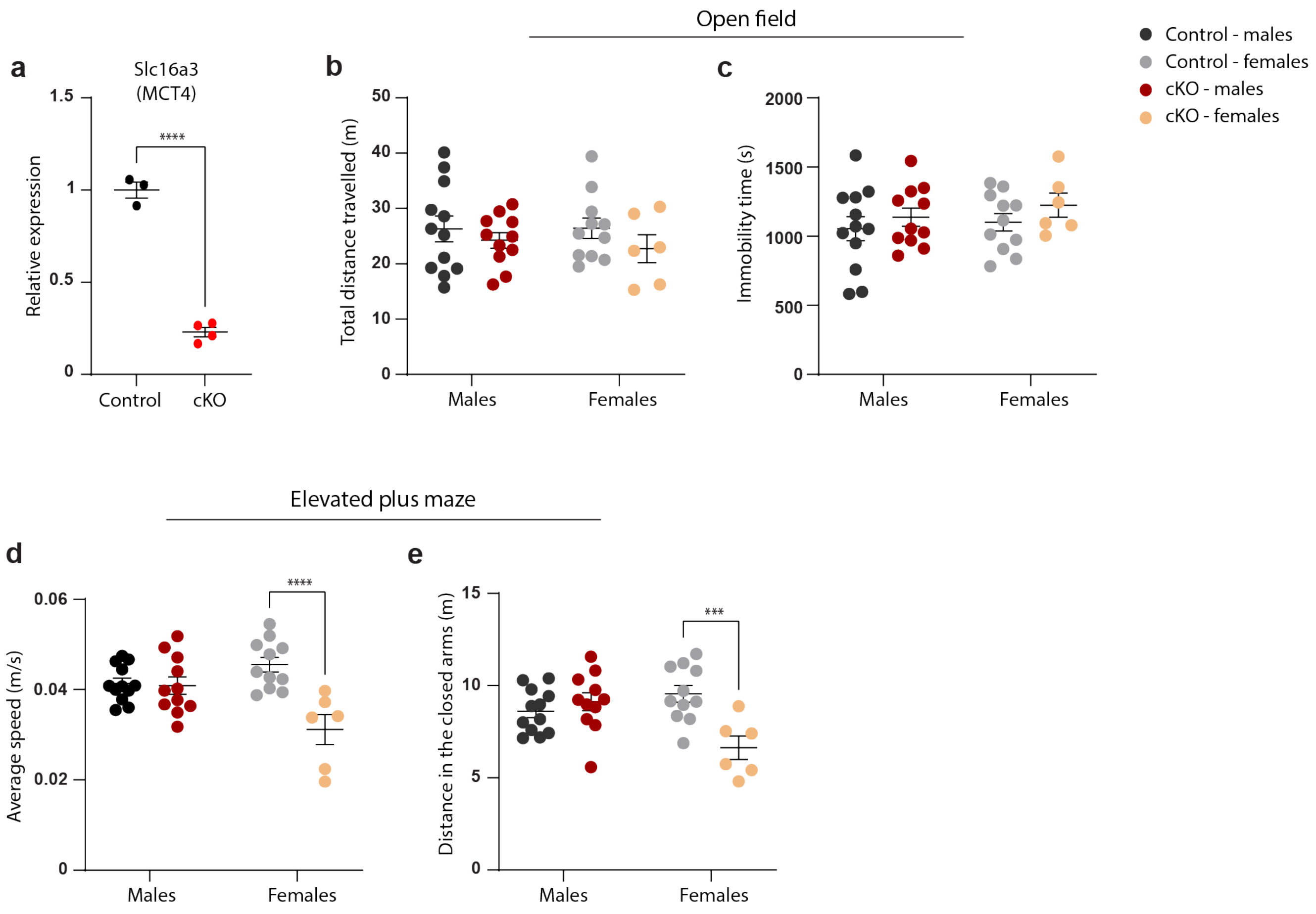

Supplementary Figure 6: MCT4 is knock out in adult microglia upon early postnatal tamoxifen injection and adult cKO mice display anxiety-like behavioral alterations

(a) Relative expression of Slc16a3 (MCT4) in microglia acutely isolated from adult mice (7-8 months). Two-tailed unpaired t-test, ****p< 0.0001. **(b-e)** Behavioral characterization performed at 7-8 months. Each data point represents one animal. Control: N=12 males; N=11 females; cKO: N=11 males; N=6 females. Statistics are calculated with two-way ANOVA followed by Sidak's post hoc multiple comparison test. Open field exploration, with **(b)** quantification of total distance travelled in the arena and **(c)** total immobility time. Elevated plus maze test, with quantification of **(d)** average speed, ****p(females) < 0.0001; **(e)** distance travelled in the closed arms, ***p(females)= 0.0006.

Uncropped blots – Supplementary Figures

Supplementary Figure 4

PSD95

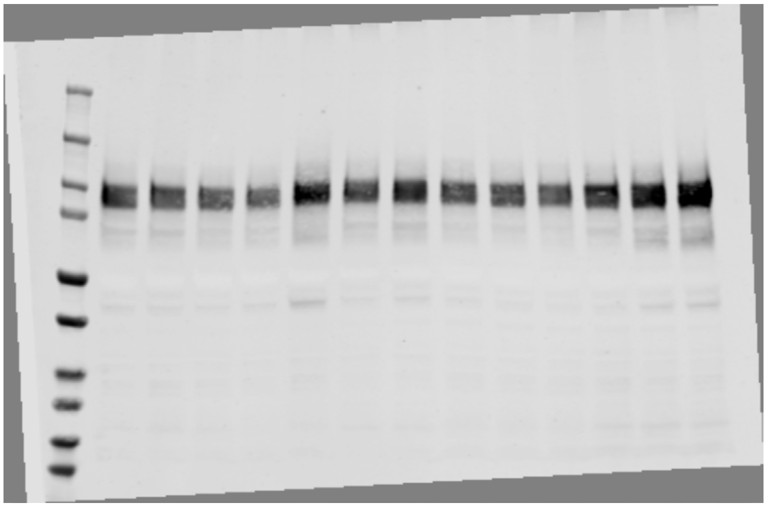

Total protein stain

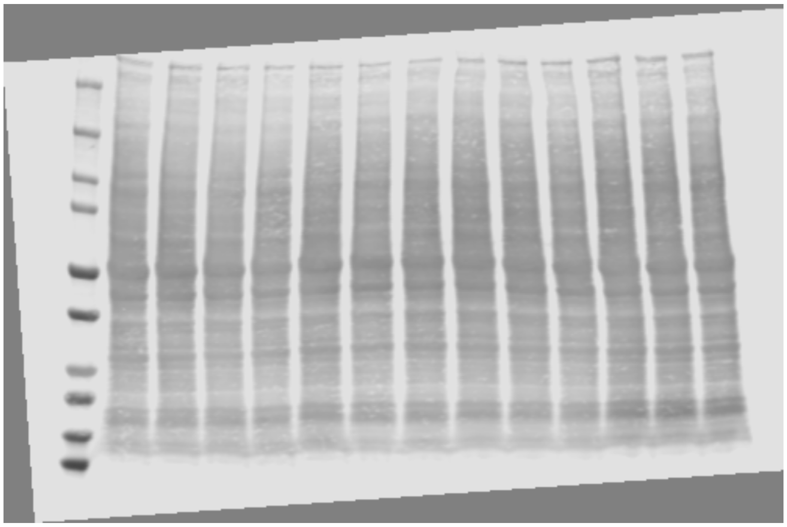

Supplement: Supplementary file 1 — Supplementary Information [file 41467_2023_41502_MOESM1_ESM.pdf]
