# Supplementary material for: Loss of microglial MCT4 leads to defective synaptic pruning and anxiety-like behavior in mice
Source: Nat Commun. 2023 Sep 16;14:5749. doi: 10.1038/s41467-023-41502-4 (PMC10505217; doi:10.1038/s41467-023-41502-4)
Supplement: Supplementary file 3 — Reporting Summary [file 41467_2023_41502_MOESM3_ESM.pdf]

nature portfolio

Corresponding author(s): Rosa Chiara Paolicelli

Last updated by author(s): Aug 10, 2023

Reporting Summary

Nature Portfolio wishes to improve the reproducibility of the work that we publish. This form provides structure for consistency and transparency in reporting. For further information on Nature Portfolio policies, see our Editorial Policies and the Editorial Policy Checklist.

Statistics

For all statistical analyses, confirm that the following items are present in the figure legend, table legend, main text, or Methods section.

n/a	Confirmed	
☐	☒	The exact sample size (*n*) for each experimental group/condition, given as a discrete number and unit of measurement
☐	☒	A statement on whether measurements were taken from distinct samples or whether the same sample was measured repeatedly
☐	☒	The statistical test(s) used AND whether they are one- or two-sided<br>*Only common tests should be described solely by name; describe more complex techniques in the Methods section.*
☐	☒	A description of all covariates tested
☐	☒	A description of any assumptions or corrections, such as tests of normality and adjustment for multiple comparisons
☐	☒	A full description of the statistical parameters including central tendency (e.g. means) or other basic estimates (e.g. regression coefficient) AND variation (e.g. standard deviation) or associated estimates of uncertainty (e.g. confidence intervals)
☐	☒	For null hypothesis testing, the test statistic (e.g. *F*, *t*, *r*) with confidence intervals, effect sizes, degrees of freedom and *P* value noted<br>*Give P values as exact values whenever suitable.*
☐	☒	For Bayesian analysis, information on the choice of priors and Markov chain Monte Carlo settings
☐	☒	For hierarchical and complex designs, identification of the appropriate level for tests and full reporting of outcomes
☒	☐	Estimates of effect sizes (e.g. Cohen's *d*, Pearson's *r*), indicating how they were calculated

Our web collection on statistics for biologists contains articles on many of the points above.

Software and code

Policy information about availability of computer code

Data collection	Confocal images were acquired by a Stellaris 5 confocal microscope (Leica), using 20x air or 63x oil objectives.<br>Electrophysiology data were acquired using Igor Pro with NIDAQ tools (WaveMetrics).<br>Metabolomics: for primary cells, chromatographic separation was carried out using a SeQuant ZIC-pHILIC column (Merck). Full scan HRMS acquisition mode (m/z 50-750) was used at resolution at 70,000 FWHM, 1 microscan, 1e6 AGC and 100 ms as maximum inject time; for hippocampal homogenates, samples were analyzed on a Thermo Vanquish UHPLC coupled to a Thermo Q Exactive mass spectrometer.<br>RT-qPCR data were collected using ViiA 7 Real-Time PCR System (Thermo Fisher Scientific).
Data analysis	The data were analyzed using Microsoft Excel and statistical analysis was performed using GraphPad Prism (version 9.3.1).<br>Images were analyzed with Fiji Software (ImageJ 1.53n) and Imaris (version 9.9.1, Bitplane).<br>Metabolomics: for primary cells, data were processed using Xcalibur (version 4.1, Thermo Fisher Scientific); for hippocampal tissues, metabolites were assigned and peak areas integrated using Maven (Princeton University).<br>Electrophysiological data were analyzed offline using Minianalysis (Synaptosoft, USA).<br>For behavioral experiments, mouse behavior was automatically tracked from the top and analyzed using the Anymaze software (version 4.99z) and with BORIS (version 8.20.4, University of Torino, Italy), as appropriate.

For manuscripts utilizing custom algorithms or software that are central to the research but not yet described in published literature, software must be made available to editors and reviewers. We strongly encourage code deposition in a community repository (e.g. GitHub). See the Nature Portfolio guidelines for submitting code & software for further information.

Data

Policy information about availability of data

All manuscripts must include a data availability statement. This statement should provide the following information, where applicable:

- Accession codes, unique identifiers, or web links for publicly available datasets
- A description of any restrictions on data availability
- For clinical datasets or third party data, please ensure that the statement adheres to our policy

The transcriptomics data (bulk RNA-seq of adult microglia cells; Pinto et al. 2012), can be downloaded from the ArrayExpress database, accession number E-MEXP-3347).
The single-cell RNA-seq data of adult hippocampal cell populations were generated for a previous study (Mattei et al. 2020). Gene count matrices are available at https://github.com/bihealth/SC-RNA-Seq-37vs4. The gene lists used to calculate module scores associated with cellular metabolic pathways have been downloaded from the Kyoto Encyclopedia of Genes and Genomes (KEGG database: https://www.genome.jp/kegg/).
The raw data of the metabolomics performed on hippocampal homogenates that have been generated in this study have been deposited in the public repository Metabolomics Workbench, under the Project ID number: ST002714.
No restrictions will be placed on materials involved in this study. All data supporting the findings of this study are available within the article and its supplementary information file, are provided in the Source Data file and can be obtained from the corresponding author upon reasonable request.

Research involving human participants, their data, or biological material

Policy information about studies with human participants or human data. See also policy information about sex, gender (identity/presentation), and sexual orientation and race, ethnicity and racism.

Reporting on sex and gender	N/A
Reporting on race, ethnicity, or other socially relevant groupings	N/A
Population characteristics	N/A
Recruitment	N/A
Ethics oversight	N/A

Note that full information on the approval of the study protocol must also be provided in the manuscript.

Field-specific reporting

Please select the one below that is the best fit for your research. If you are not sure, read the appropriate sections before making your selection.

☒ Life sciences ☐ Behavioural & social sciences ☐ Ecological, evolutionary & environmental sciences

For a reference copy of the document with all sections, see nature.com/documents/nr-reporting-summary-flat.pdf

Life sciences study design

All studies must disclose on these points even when the disclosure is negative.

Sample size	No statistical method was used to predetermine sample size. Sample size was chosen based on our previous studies using the same types of assays, as well as published literature (Paolicelli et al. 2011; Zhan et al 2014; Paolicelli et al. 2017; Filipello et al. 2018).<br>In vitro studies used a standard of at least N=3 independent experiments, within each 2 or 3 technical replicates were always used and averaged.<br>For hippocampal tissue metabolomics, N=9-10 mice per group were included.<br>For electrophysiological experiments, slices from N=2-5 control mice and N=2-4 cKO mice were used.<br>Histology and 3D cell reconstructions were performed on 3-5 mice per genotype.<br>Kainic acid (KA) in vivo studies were performed on 6 mice per genotype (3 males and 3 female per group).<br>Sample sizes for behavioral experiments were determined based on the current standard, as the minimum amount of mice required to detect significance with an alpha rate set at .05 in a standardly powered experiment.
Data exclusions	Outliers identification test was performed usign GraphPad Prism (version 9.3.1).
Replication	All the experiments were repeated at least three times, with the exception of the in vitro assay for LysoTracker and DQ-BSA quantification in control and cKO microglia, which were performed twice.<br>Independent experiments were always performed with at least 2 technical replicates.<br>All experiments were reproduced to reliably support conclusions stated in the manuscript.

Randomization	Animals were assigned randomly to experimental groups.
Blinding	Animals were treated blindly to the experimenters. Data analysis for both in vitro and in vivo experiments was performed blindly to the experimenters.

Reporting for specific materials, systems and methods

We require information from authors about some types of materials, experimental systems and methods used in many studies. Here, indicate whether each material, system or method listed is relevant to your study. If you are not sure if a list item applies to your research, read the appropriate section before selecting a response.

Materials & experimental systems

n/a	Involved in the study
☐	☒ Antibodies
☒	☐ Eukaryotic cell lines
☒	☐ Palaeontology and archaeology
☐	☒ Animals and other organisms
☒	☐ Clinical data
☒	☐ Dual use research of concern
☒	☐ Plants

Methods

n/a	Involved in the study
☒	☐ ChIP-seq
☒	☐ Flow cytometry
☒	☐ MRI-based neuroimaging

Antibodies

Antibodies used	The following antibodies were used in this work, at the stated concentrations: MCT4, RRID: AB_2189333, cat. sc-50329, Santa Cruz BioTechnology (WB 1:500); IBA1, RRID: AB_839504, cat. 019-19741, FUJIFILM Wako (IF 1:1000, lot CAF6806); CD68, RRID: AB_322219, cat. MCA1957, Bio-Rad (IF 1:400, lot 155083); Synapsin1, RRID: AB_2619772, cat. 106011, Synaptic Systems (WB 1:1000, lot 1-28); Homer1, RRID: AB_887730, cat. 160003, Synaptic Systems (WB 1:1000, IF 1:200, lot 3-61); VGAT, RRID: AB_887872, cat. 131011, Synaptic Systems (WB 1:1000, lot 1-91); Gephyrin, RRID: AB_887719, cat 147111, Synaptic Systems (WB 1:1000, lot 1-25); GluA1, RRID: AB_2113602, cat. AB1504, Merck Millipore (WB 1:1000); GluA2, RRID: AB_2533058, cat. 32-0300, Thermo Fisher Scientific (WB 1:250); Synapsin1, RRID: AB_2721082, cat. 106104, Synaptic Systems (IF 1:200, lot 1-4); VGLUT1, RRID: AB_887880, cat. 135311, Synaptic Systems (WB 1:1000, lot 1-7); PSD95, RRID: AB_2092365, cat. MAB1596, Merck Millipore (WB 1:1000, IF 1:200, lot 3845700); LAMP1, RRID: AB_2134500, cat. 1D4B-c, Developmental Studies Hybridoma Bank (IF 1:100, lot 1-6-22).
Validation	The MCT4 antibody was KO validated in-house, using MCT4 KO microglia. The Wako IBA1 antibody has been cited in 3838 studies. The Biko-rad CD68 antibody has been cited in more than 200 studies. The following antibodies are KO validated: Synapsin1 cat no 106011, VGAT cat no 131011, Gephyrin cat no 147111, Synapsin1 cat no 106104, VGLUT1 cat no 135311.<br>The GluA2 antibody, cat no 32-0300, was verified by cell treatment by the manufacturer to ensure that it binds to the antigen stated.

Animals and other research organisms

Policy information about studies involving animals; ARRIVE guidelines recommended for reporting animal research, and Sex and Gender in Research

Laboratory animals	This study is based on the use of mice as animal models. Experiments have been performed at 2 weeks of age (P15) and in adulthood (7 months). The following strains have been crossed and used: B6.129P2(Cg)-Cx3cr1tm2.1(cre/ERT2)Litt/WganJ mice (Cx3cr1CREERT2; No: 021160, The Jackson Laboratory); C57Bl/6.MCT4tm1flox mice (MCT4flox; kindly provided by Prof. Luc Pellerin); B6.Cg-Gt(ROSA)26Sortm14(CAG-tdTomato)Hze/J mice (TdTomatoflox; No: 007914, The Jackson Laboratory).
Wild animals	No wild animals were used in this study.
Reporting on sex	For most of the experiments both sexes have been used, as reported by color-code in the figures, in figure legends, and in the text.
Field-collected samples	No field-collected samples were used in this study.
Ethics oversight	All animal experiments were authorized by the "Service de la consommation et des Affaires vétérinaires" (SCAV) of the Canton de Vaud in Switzerland, and approved by the ethical committee.

Note that full information on the approval of the study protocol must also be provided in the manuscript.
