# Supplementary material for: Loss of microglial MCT4 leads to defective synaptic pruning and anxiety-like behavior in mice
Source: Nat Commun. 2023 Sep 16;14:5749. doi: 10.1038/s41467-023-41502-4 (PMC10505217; doi:10.1038/s41467-023-41502-4)

Figure 2b

Antibody: MCT4

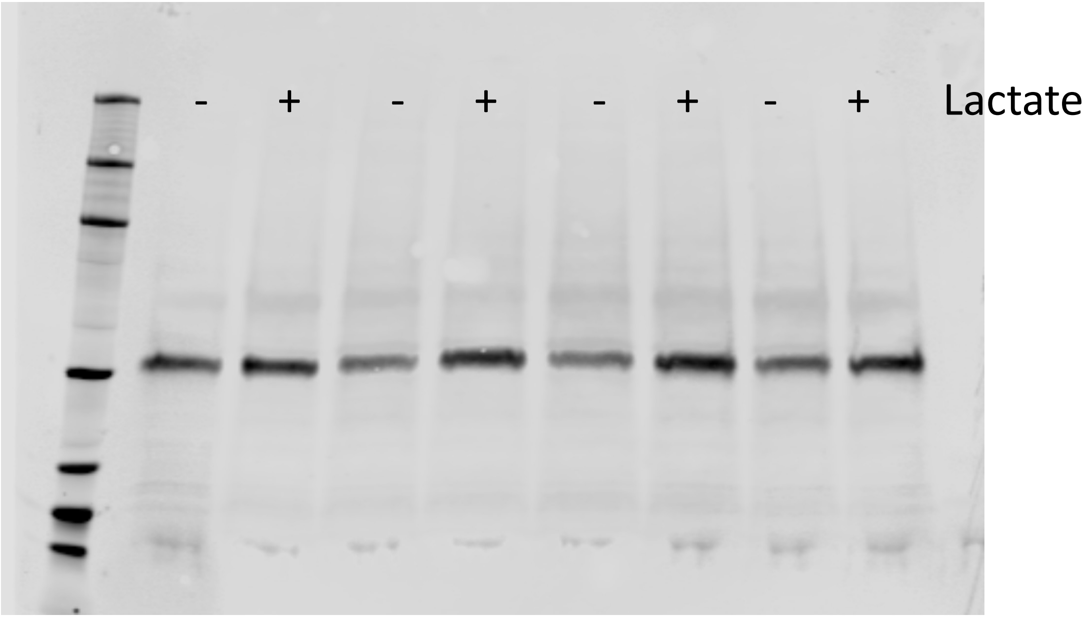

Total protein stain

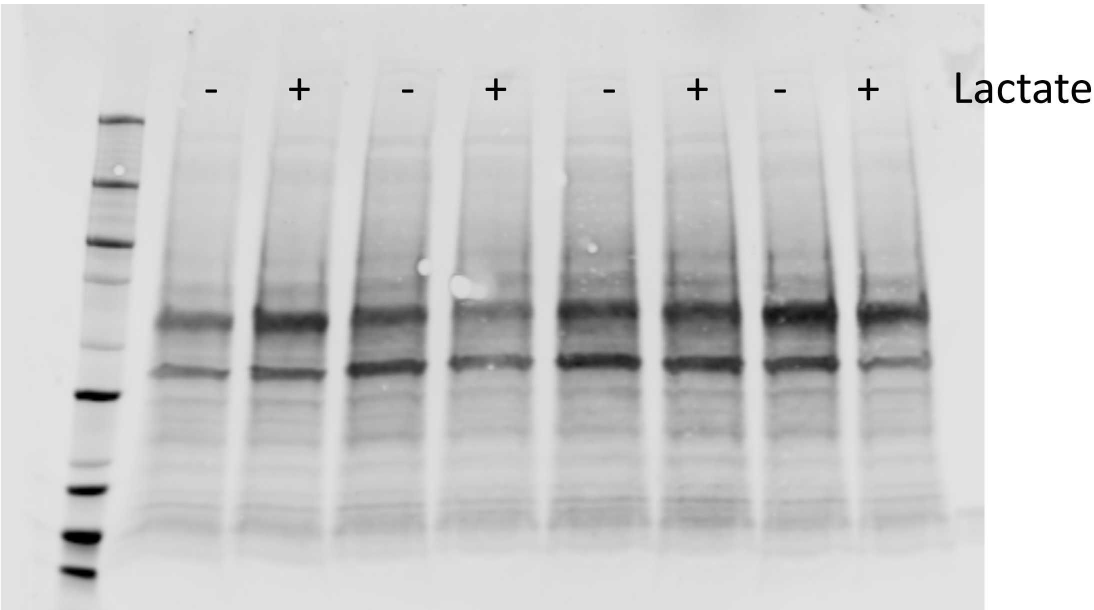

Figure 2e

Antibody: MCT4

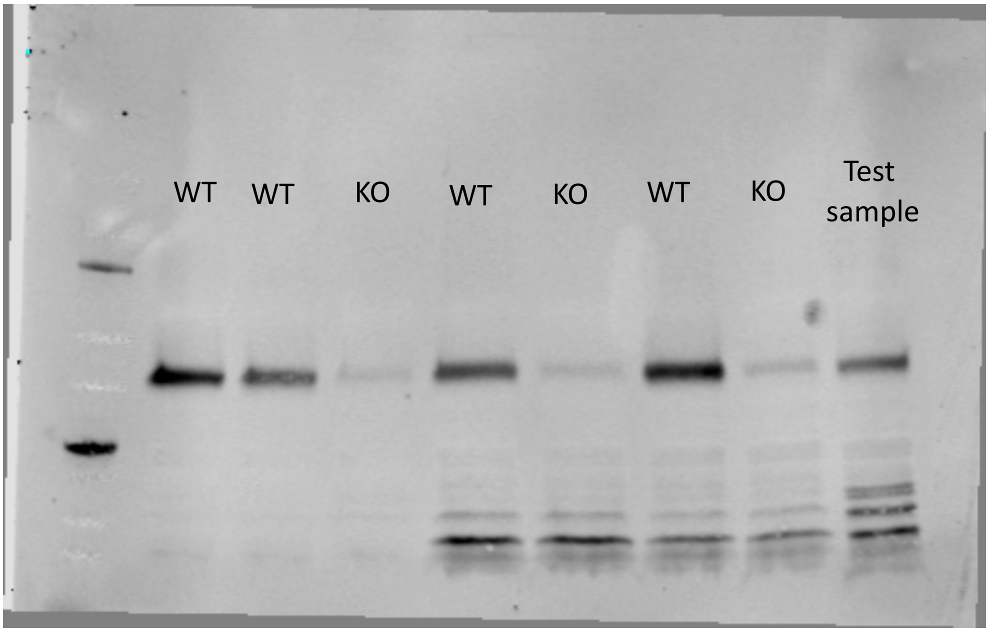

Total protein stain

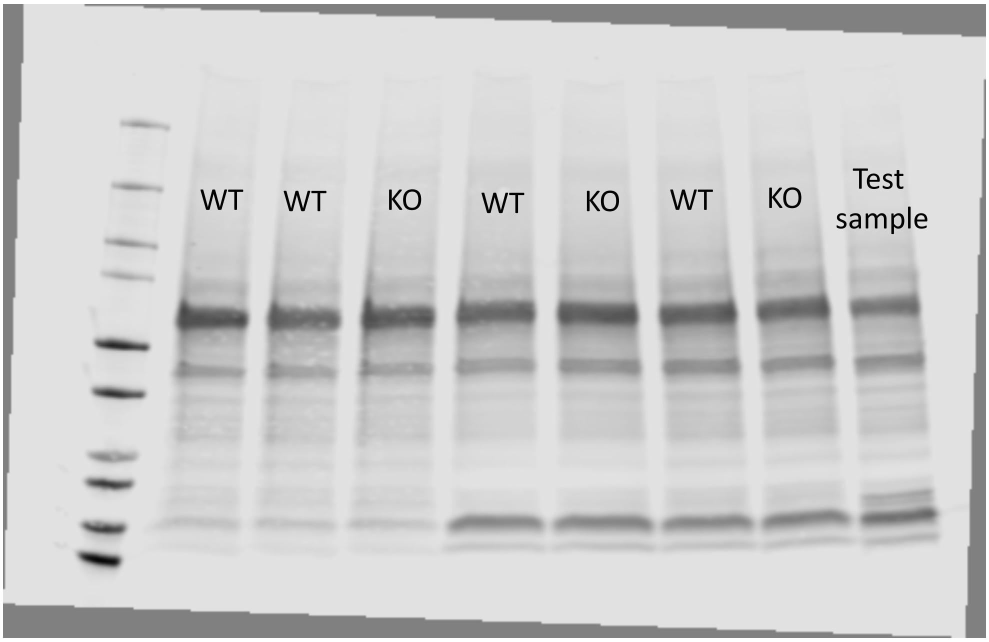

Figure 3h

Antibody: MCT4

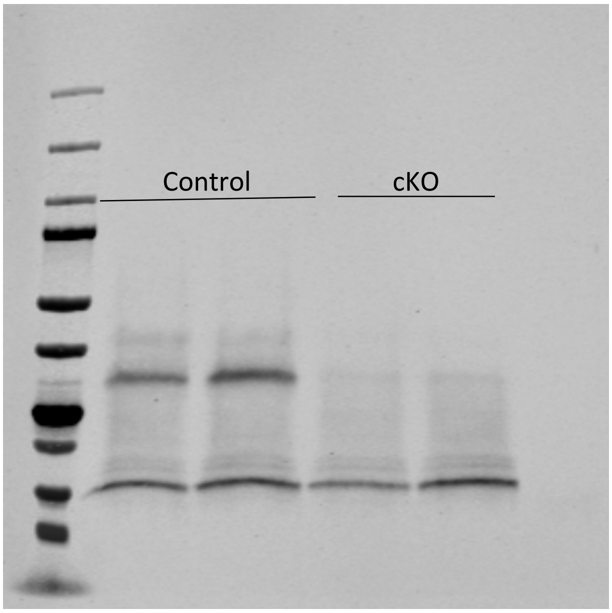

Total protein stain

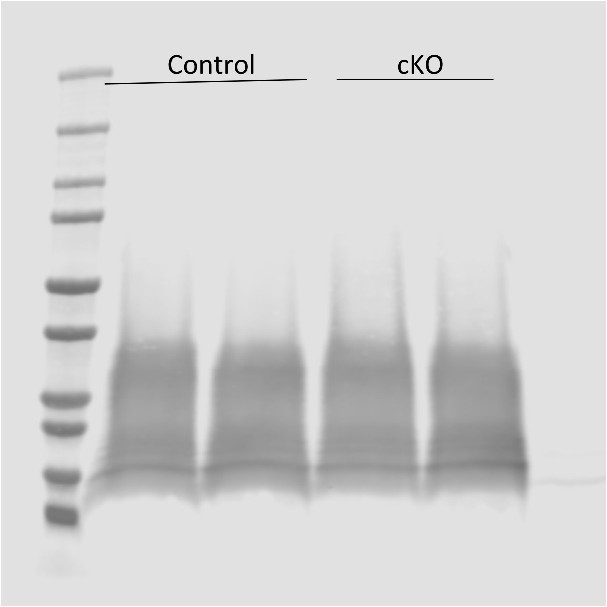

Figure 4a

Antibody: Gephyrin

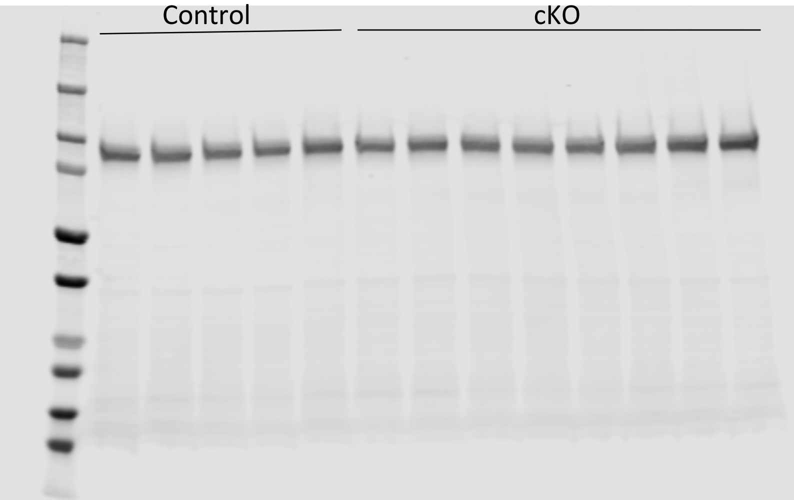

Antibody: VGAT

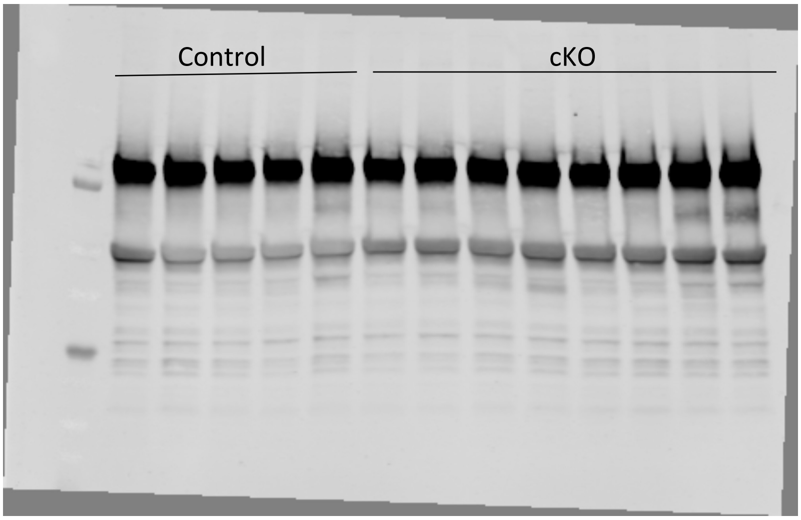

Total protein stain

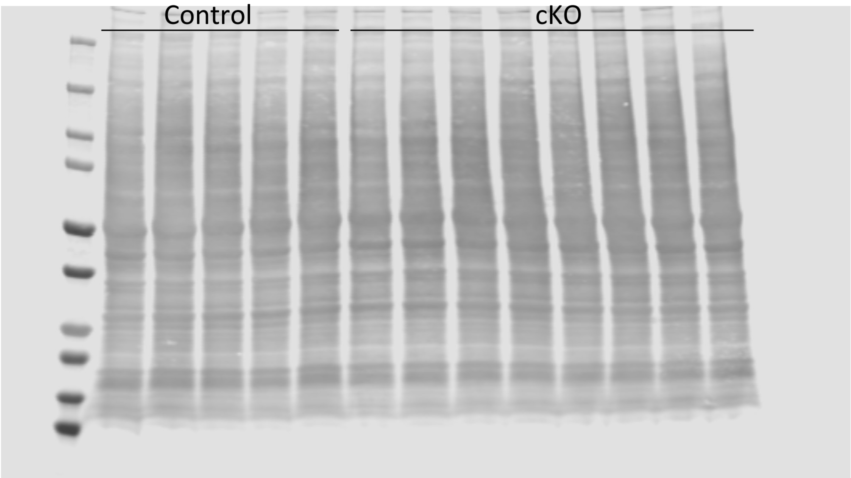

Figure 4a

Antibody: GluA1

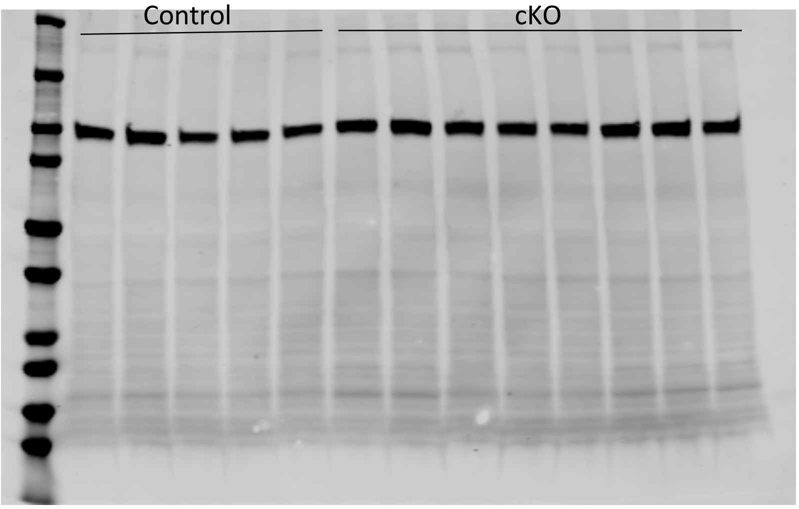

Antibody: GluA2

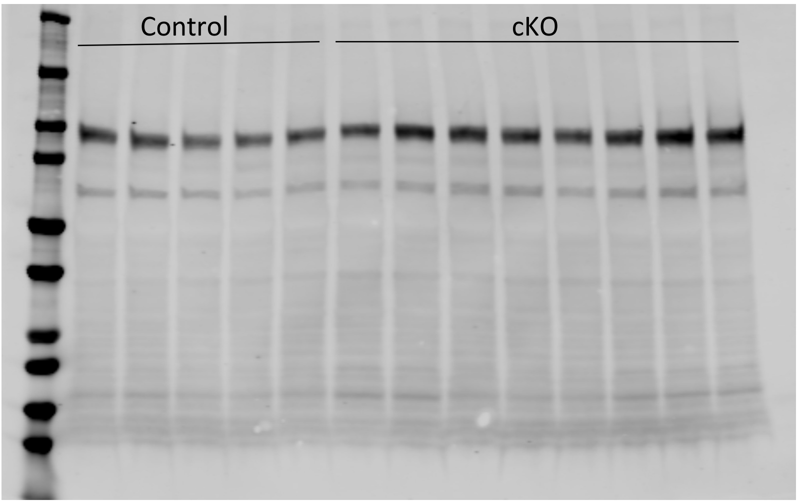

Total protein stain

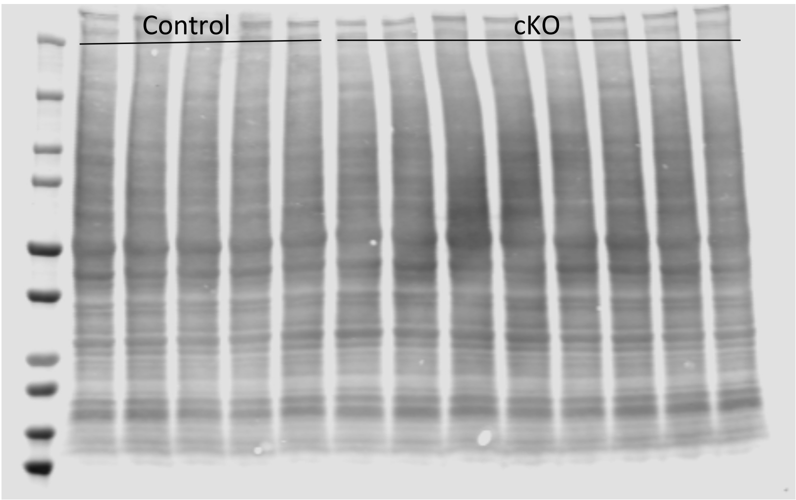

Figure 4a

Antibody: Homer

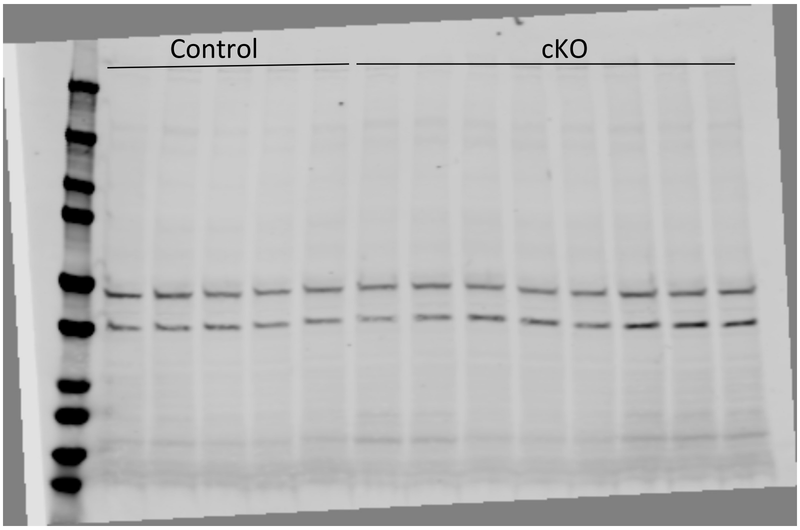

Antibody: Synapsin

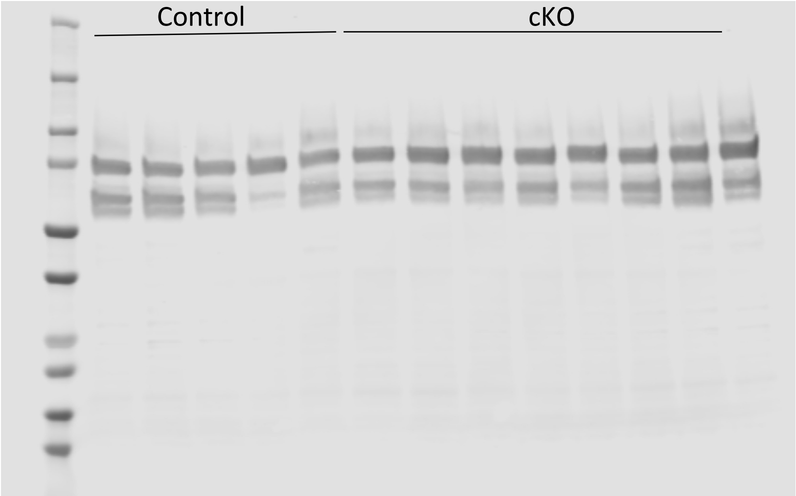

Total protein stain

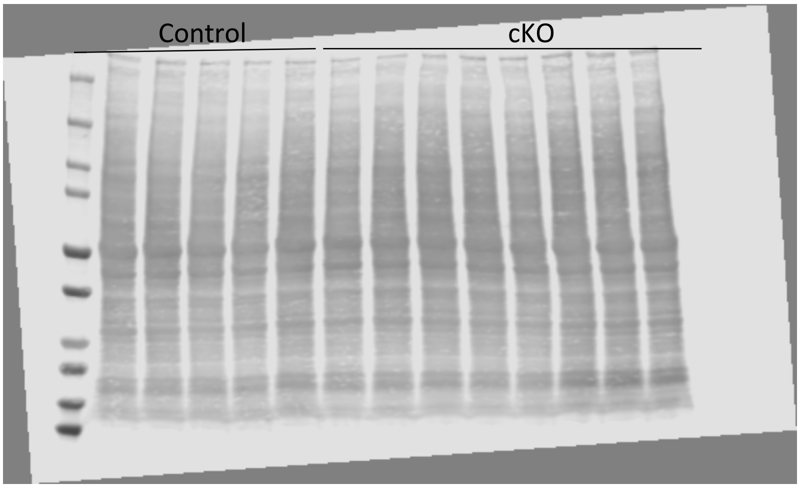

Figure 7a

Antibody: Gephyrin

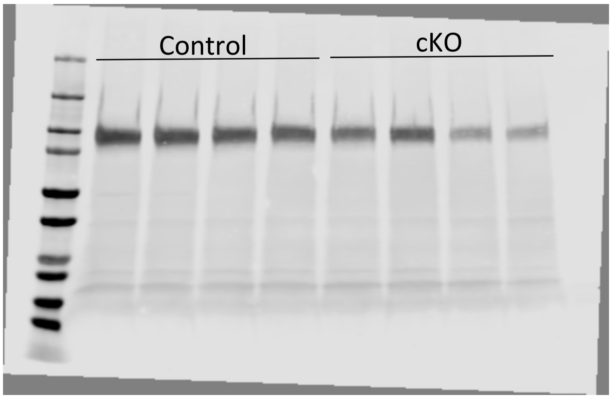

Antibody: VGAT

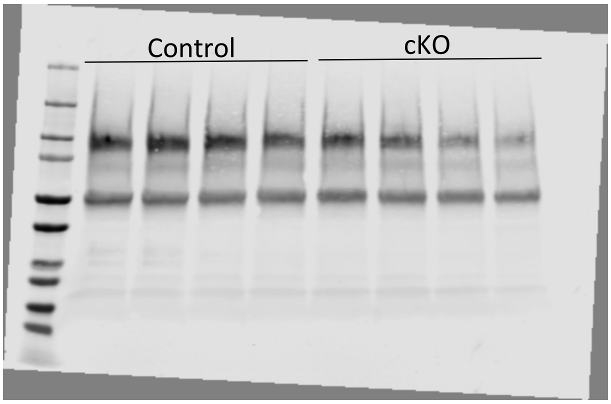

Antibody: GluA2

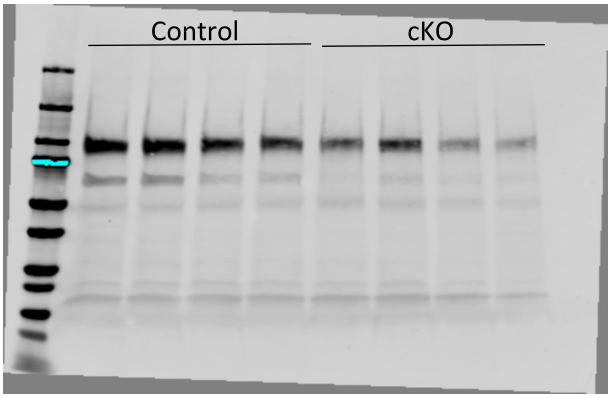

Total protein stain

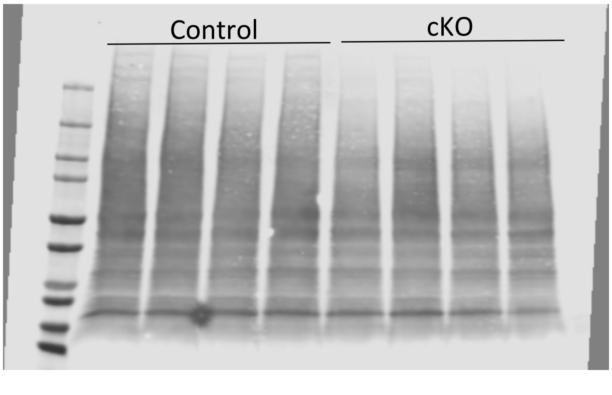

Figure 7a

Antibody: VGLUT1

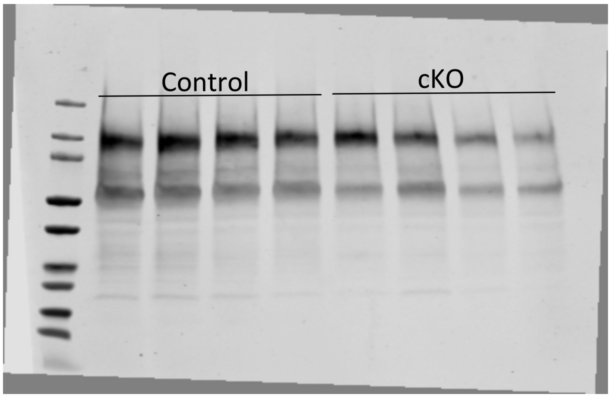

Antibody: PSD95

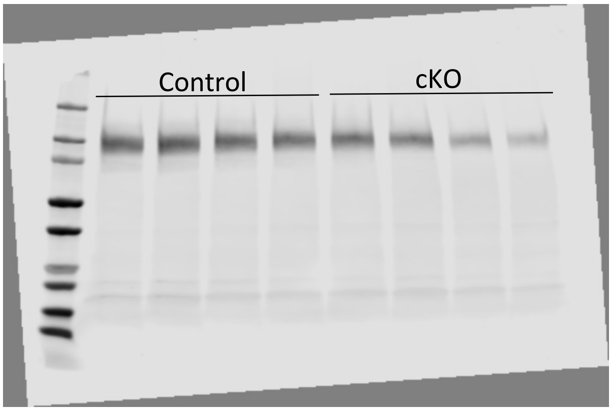

Antibody: GluA1

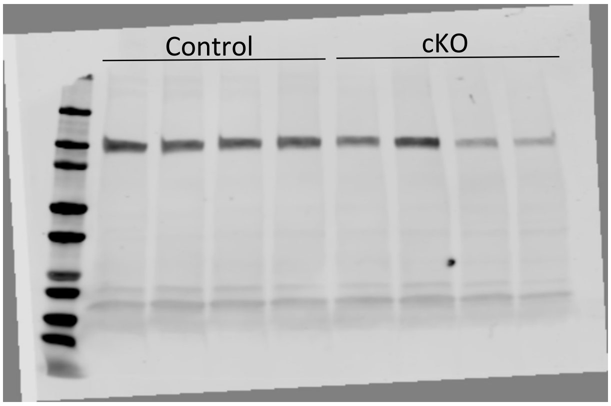

Total protein stain

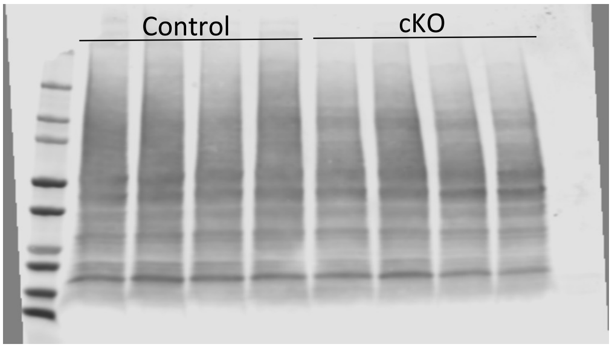

Supplement: Supplementary file 4 — Source Data [file 41467_2023_41502_MOESM4_ESM.zip › Source Data Blots.pdf]
